# Preparation of Chitin Nanofibers and Natural Rubber Composites and Their Triboelectric Nanogenerator Applications

**DOI:** 10.3390/ma17030738

**Published:** 2024-02-03

**Authors:** Kattaliya Petchnui, Teerayut Uwanno, Mayuree Phonyiem Reilly, Chinathun Pinming, Alongkot Treetong, Visittapong Yordsri, Nutthanun Moolsradoo, Annop Klamcheun, Winadda Wongwiriyapan

**Affiliations:** 1 College of Materials Innovation and Technology, King Mongkut’s Institute of Technology Ladkrabang, Chalongkrung Rd., Ladkrabang, Bangkok 10520, Thailand; kattaliya.pang@gmail.com (K.P.); teerayut.uw@kmitl.ac.th (T.U.); mayuree.ph@kmitl.ac.th (M.P.R.); chinathun.aron@gmail.com (C.P.); 2 National Nanotechnology Center (NANOTEC), National Science and Technology Development Agency (NSTDA), 111 Thailand Science Park, Paholyothin Rd., Klong Nueng, Klong Luang, Pathum Thani 12120, Thailand; alongkot@nanotec.or.th (A.T.); annop@nanotec.or.th (A.K.); 3 National Matal and Materials Technology Center (MTEC), National Science and Technology Development Agency (NSTDA), 111 Thailand Science Park, Paholyothin Rd., Klong Nueng, Klong Luang, Pathum Thani 12120, Thailand; visittay@mtec.or.th; 4 Department of Production Technology Education, Faculty of Industrial Education and Technology, King Mongkut’s University of Technology Thonburi, 126 Pracha Uthit Rd., Thung Khru, Bangkok 10140, Thailand; nutthanun.moo@kmutt.ac.th

**Keywords:** triboelectric nanogenerator, chitin nanofiber, natural rubber

## Abstract

Triboelectric nanogenerators (TENGs) have gained significant attention as promising energy-harvesting devices that convert mechanical energy into electrical energy through charge separation induced by friction and electrostatic induction. In this study, we explore the utilization of biowaste shrimp shell-extracted chitin nanofiber (ChNF) as a viable eco-friendly material for TENG applications. Composite materials were prepared by incorporating ChNF into natural rubber (NRL) at loading levels of 0.1 and 0.2 wt% (NRL/ChNF) to form the TENG triboelectric layer. ChNFs with a uniform width of approximately 10–20 nm were successfully extracted from the shrimp shells through a simple mechanical procedure. The NRL/ChNF composites exhibited enhanced mechanical properties, as evidenced by a higher Young’s modulus (3.4 GPa) compared to pure NRL. Additionally, the NRL/ChNF composites demonstrated an increased dielectric constant of 3.3 at 0.1 MHz. Moreover, the surface potential difference of NRL increased from 0.182 V to 1.987 V in the NRL/ChNF composite. When employed as the triboelectric layer in TENG, the NRL/ChNF composites exhibited significant improvement in their output voltage, with it reaching 106.04 ± 2.3 V. This enhancement can be attributed to the increased dielectric constant of NRL/ChNF, leading to enhanced charge exchange and charge density. This study presents a straightforward and environmentally friendly technique for preparing sustainable natural materials suitable for energy-harvesting devices.

## 1. Introduction

Energy scavenging through sustainable pathways has gained significant attention as a means to address global energy challenges and enable practical solutions for powering electronic devices and sensors in emerging technologies. Triboelectric nanogenerators (TENGs) have emerged as promising devices capable of harvesting mechanical energy through contact triboelectrification and electrostatic induction [1,2]. Current research efforts focus on enhancing TENG power output by increasing the contact area for triboelectrification and modifying the material properties of the contacting surfaces [3,4,5,6,7,8,9]. Most TENGs developed to date have utilized synthetic polymers due to their excellent dielectric properties and mechanical robustness [10,11]. While synthetic polymers like polypropylene and polytetrafluoroethylene are widely used in medical applications, their integration into TENGs for certain biomedical applications can be limited by specific biocompatibility requirements, particularly in scenarios involving prolonged direct contact with biological tissues. Consequently, there is a need to explore TENGs employing natural materials, harnessing wasted resources to harvest ambient mechanical energy and addressing practical societal requirements [12,13].

Chitin, a cellulose analogue characterized by a (1,4)-β-N-acetyl glycosaminoglycan repeating structure, is the second most abundant biopolymer following cellulose. It is primarily found in the exoskeletons of shellfish and insects, as well as the cell walls of mushrooms. Despite chitin’s semicrystalline nature, nano-sized fibrillar morphology, and exceptional material properties, a significant portion of chitin is discarded as industrial waste. Therefore, it becomes crucial to harness the potential of chitin as an environmentally friendly and sustainable material. Chitin nanofibers (ChNFs) with diameters less than 100 nm and aspect ratios exceeding 100 possess an exceptionally high surface-to-volume ratio, resulting in the formation of highly porous meshes with distinct properties compared to micro-sized fibers [14,15,16]. The preparation of nanofibers holds significant importance due to their unique dimensional, optical, mechanical, and other characteristics, which enable the exploration of numerous advanced material properties and hold promise for various applications. Considering the presence of amino groups with excellent electron-donating functionality, chitin nanofibers (ChNFs) can be regarded as ideal tribo-positive polymers for fabricating TENGs. The abundant amino groups in ChNFs make them prone to electron loss and easy to achieve a positive charge, making ChNF a candidate material for polymer-based eco-friendly TENGs [17,18,19].

Natural rubber (NRL), also known as polyisoprene, is a natural polymer that is widely utilized for its flexibility and strength in a variety of applications. Numerous NRL products, such as car tires, shoe insoles, and mattresses, are routinely subjected to mechanical energy sources. NRL is characterized by a slightly negative polarity in the triboelectric series, which qualifies it as an appropriate material for triboelectric applications. Recent studies have focused on engineering NRL composites by incorporating various fillers to improve their properties [20,21,22,23,24,25]. The fabrication of NRL and ChNF composites (NRL/ChNF) for use in TENGs has shown promise in enhancing power output and advancing eco-friendly TENG technology. Thus, to develop eco-friendly TENGs based on NRL/ChNF, it is necessary to study the methods of disintegrating chitin into ChNF, as well as the preparation of NRL/ChNF and their properties.

In this work, ChNFs with a uniform width were successfully extracted from shrimp shells using a simple mechanical procedure after removing proteins and minerals. These ChNFs were then incorporated into NRL material, forming NRL/ChNF composites used as triboelectric materials to convert mechanical energy into electricity. The Young’s modulus, dielectric constant, and TENG output performance of the NRL/ChNF composites were investigated. The composites exhibited a significant improvement in output voltage, with it reaching 106.04 ± 2.3 V. This enhancement can be attributed to the increased surface area, improved recovery time, and higher dielectric constant of the NRL/ChNF composites.

## 2. Materials and Methods

### 2.1. Preparation of Chitin Nanofibers

A fresh shrimp exoskeleton was collected from a local market in Thailand and used as the starting material. The exoskeleton was cleaned and dried for further chemical and mechanical treatments. 

The extraction process for minerals, pigments, and proteins from the exoskeleton was conducted in the following sequential manner. Typically, 50 g of shrimp exoskeleton was initially demineralized by immersing it in 200 mL of 2 M HCl for a duration of 2 h. Subsequently, it was placed in a solution of 200 mL of 1 M NaOH to facilitate the removal of proteins. Finally, the exoskeleton was treated with 95% ethanol and stirred for 30 min to eliminate pigments, resulting in the purified chitin.

Following ethanol treatment, the chitin was rinsed with de-ionized (DI) water and suspended in 900 mL of DI water. The resulting colloidal suspension was then blended using a powerful kitchen blender and subjected to high-pressure microfluidization (HPM) using a M-110P Microfluidizer (Microfluidics Ind., Westwood, MA, USA). The HPM process consisted of two stages: the first at 1600 bar and the second at 1950 bar. The optimal cycles for the first and second stages (X,Y) were investigated using the following combinations: (5,5), (5,10), (10,5), and (10,10), where X and Y represent the number of cycles for the first and second stages, respectively.

### 2.2. Preparation of Natural Rubber and Chitin Nanofiber Composites

NRL was filtered with a 300-mesh sieve before use. The desired amount of filtered NRL and ChNF suspension was mixed, and subsequently, vulcanizing agents were added under magnetic stirring for 10 min to obtain homogeneous NRL/ChNFs suspensions. The vulcanizing agents included S (1 phr), ZnO (0.8 phr), C_15_H_24_O (1 phr), and C_10_H_20_N_2_S_4_Zn (0.4 phr). Then, the suspension was vulcanized at 80 °C for 1 h in an oven. Then, the suspension was poured into a plastic mold with a thickness of approximately 200 μm and solidified and vulcanized at 80 °C for 24 h. Finally, the prepared NRL/ChNF composites were marked as ChNF0.05, ChNF0.1, and ChNF0.2 based on the different loadings of ChNFs (in wt%).

### 2.3. Characterization

The morphologies of ChNF and NRL/ChNF were characterized using a field-emission scanning electron microscope (FE-SEM, JSM-7800F PRIME, JEOL Ltd., Tokyo, Japan) with an accelerated voltage of 3 kV, and the morphologies of ChNF and NRL/ChNF were studied. The histogram of the diameters of the ChNF was plotted using the ImageJ program (version 1.53v), which randomly measured the diameters of 100 ChNF samples from SEM images. The functional groups of the samples were recorded using a Fourier transform infrared (FT-IR) spectrometer (Spectrum Two FT-IR Spectrometer, PerkinElmer Inc., Llantrisant, UK) at 400–4000 cm^−1^. The capacitance of the samples was measured using an LCR meter (E4980AL, Keysight Technologies, Santa Rosa, CA, USA) at an AC signal frequency of 1 MHz. To characterize the dielectric constant of ChNF, a ChNF membrane was prepared. This ChNF membrane was prepared through a simple vacuum drying process for the ChNF suspension after HPM treatment. The topography and surface potential difference (SPD) of the NRL/ChNF composites were analyzed using Kelvin probe force microscopy (KPFM) in hover mode in an atomic force microscopy (AFM) system (NanoWizard 3, JPK Instruments, Berlin, Germany). The utilized cantilever was a Bruker AFM Probe SCM-PIT-V2, featuring a tip radius of 25 nm, a resonance frequency of 75 kHz, and a spring constant of 3 N/m. This cantilever was coated with Pt/Ir. For topography recording, an intermittent contact mode was employed. The settings for KPFM measurement included a hover height of 50 nm, a scan area of 2.5 µm^2^, a scan speed of 1 Hz, and a resolution of 64 × 64 pixels. The Young’s moduli of ChNF and NRL/ChNF were evaluated using an AFM system (NanoWizard 3, JPK Instruments, Berlin, Germany) in quantitative imaging mode. Cantilevers served as soft nanoindenters. The utilized cantilever was an Olympus AFM probe OMCL-AC160TS-R3, with a resonance frequency of 301.1 kHz and a spring constant of 20.95 N/m. The settings for the AFM measurement included a force of 30 nN, a scan area of 10 µm², and a resolution of 256 × 256 pixels. The Young’s modulus value was computed using the Hertz model.

### 2.4. Fabrication of Triboelectric Nanogenerators Based on Natural Rubber and Chitin Nanofiber Composites 

TENGs were fabricated to work in dielectric-to-dielectric mode with a vertical contact-separation structure. The polyethylene terephthalate with an indium tin oxide film coating (PET/ITO) was used as a positive electrode, and the NRL/ChNF composites attached to another PET/ITO (hereafter referred to as NRL/ChNF-PET/ITO) were used as a negative electrode. Thus, the contact materials were PET and NRL/ChNF. The contact areas of the two surfaces were 1 × 1 cm^2^. The NRL/ChNF-PET/ITO, in which the NRL/ChNF composite was used as a triboelectric layer, was prepared as follows, as depicted in Figure S1. A 1 × 1 cm^2^ area for the NRL/ChNF triboelectric layer was marked on the ITO side of the PET/ITO sheet and secured with Kapton tape. Subsequently, the NRL/ChNF suspension was poured into the specified area and dried at 80 °C for 24 h in an oven. Schematic views of the NRL/ChNF triboelectric layer preparation process and the TENG device are shown in Figure S1.

Conducting wires were connected to the two ITO electrodes through an external circuit for measurement. The two electrification layers are subjected to contact-separation motions provided by a linear motor, and the output voltage signals of the as-fabricated TENG were measured by an oscilloscope (RIGOL).

## 3. Results and Discussion

**Figure 1:**
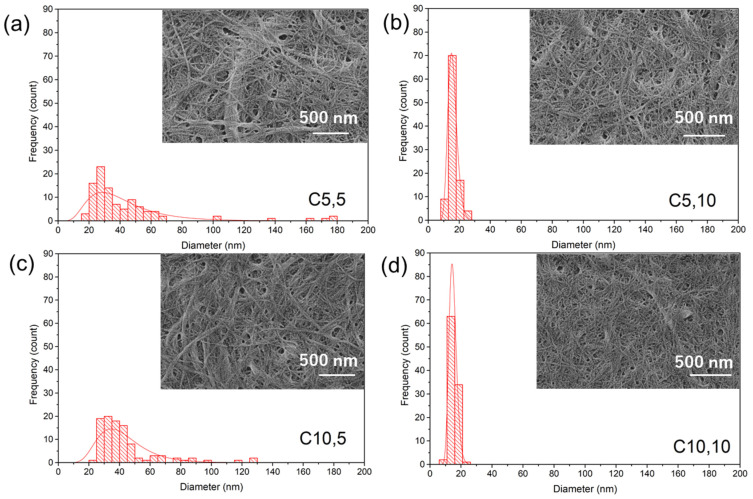
SEM images of ChNFs obtained from different HPM cycle numbers: (**a**) C5,5, (**b**) C5,10, (**c**) C10,5, and (**d**) C10,10. The images are accompanied by their respective histogram analyses of ChNF diameters.

Figure 1a–d displays SEM images of ChNFs obtained from various HPM cycle numbers (C5,5; C5,10; C10,5; and C10,10, respectively), accompanied by their respective histogram analyses of ChNF diameter. Each image reveals a nano-scale fibrous structure interwoven into a network. C5,5 signifies that the sample was subjected to five cycles in the first stage and another five cycles in the second HPM stage. The mesh-like formation implies a level of dispersion and ChNF separation, but there is noticeable overlapping and clustering in certain regions.

For C5,10, which underwent five cycles in the initial stage and ten in the subsequent stage, the fibrous network appears denser and exhibits a more consistent distribution than C5,5. The extended cycles in the second stage seemingly enhance ChNF dispersion and individualization. With C10,5, entailing ten cycles in the first stage and five in the second, some clustering is visible, yet the overall distribution seems more homogeneous than in C5,5. This suggests that the preliminary 10 cycles are pivotal in breaking down aggregates. C10,10, which was subjected to 10 cycles in both stages, presents a fairly uniform fibrous structure, making individual ChNFs more distinguishable than in other samples. This indicates optimal ChNF dispersion and separation when both stages have the maximum cycle count.

Furthermore, the histograms of C5,5 and C10,5 present wider ChNF diameter distributions, indicating a combination of both thinner and thicker ChNFs. The overlapping observed in the SEM images may contribute to the varied diameter ranges. Conversely, the histograms for C5,10 and C10,10 exhibit pronounced peaks, signifying a more consistent ChNF diameter. This aligns with the superior dispersion noted in the SEM images under these conditions.

From these observations, the HPM process demonstrates efficacy in dispersing and differentiating ChNFs. The cycle count in each phase significantly influences ChNF structure and diameter diversity. Elevating the cycle count in the second stage, as observed when comparing C5,5 (a) with C5,10 (b), optimizes ChNF dispersion, resulting in a uniform fibrous structure and consistent ChNF diameter. The preliminary cycles in the initial stage are also vital. As seen when comparing C5,5 (a) with C10,5 (c), even with identical cycles in the second stage, more cycles in the first stage led to enhanced dispersion and a homogeneous structure. Under our experimental conditions, the ideal condition for achieving well-distributed ChNFs with a consistent diameter seems to be C10,10, where both stages experience the maximum cycle number. The diameter range obtained aligns with results from previous studies using different methods (i.e., grinding treatment, a high-pressure water jet, and electrospinning) [16,26,27].

**Figure 2:**
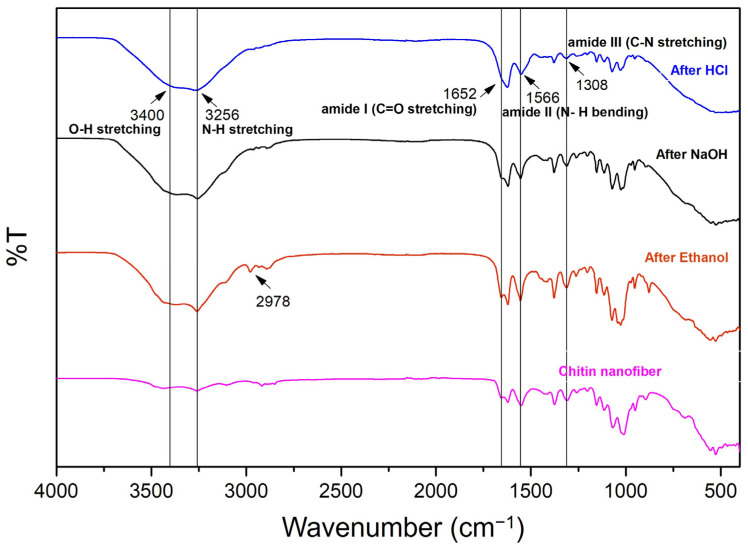
FTIR spectra of shrimp shell-extracted chitin and ChNFs.

Figure 2 shows FTIR spectra depicting the characteristic peaks associated with ChNFs (C10,10), which have undergone a series of treatments, specifically using HCl, NaOH, and ethanol. The HCl treatment primarily aims to demineralize the shrimp shell, removing calcium carbonate. The NaOH treatment is employed to deproteinize the chitin, removing proteins from the chitin. Ethanol treatment is generally used to defat and further purify the chitin. The FTIR spectral data provide insight into the chemical functionalities present in the treated chitin samples. The following functional groups corresponding to transmittance peaks are observed: O-H stretching (~3400 cm^−1^), which is associated with the hydroxyl groups present in chitin, and N-H stretching (~3256 cm^−1^), which suggests the presence of amine groups in the chitin structure. Amide I (C=O stretching) (~1652 cm^−1^) provides evidence for the presence of acetylated units in chitin. Amide II (N-H bending) (~1566 cm^−1^) further confirms the presence of amide linkages in the structure. Amide III (C-N stretching) (~1308 cm^−1^) is representative of the C-N stretching vibrations within the amide groups of chitin. C-H stretching (~2978 cm^−1^) suggests the presence of methyl and methylene groups in chitin. After simple mechanical defibrillation of chitin to ChNFs, there were no obvious changes in the peaks of the FTIR spectra. 

**Figure 3:**
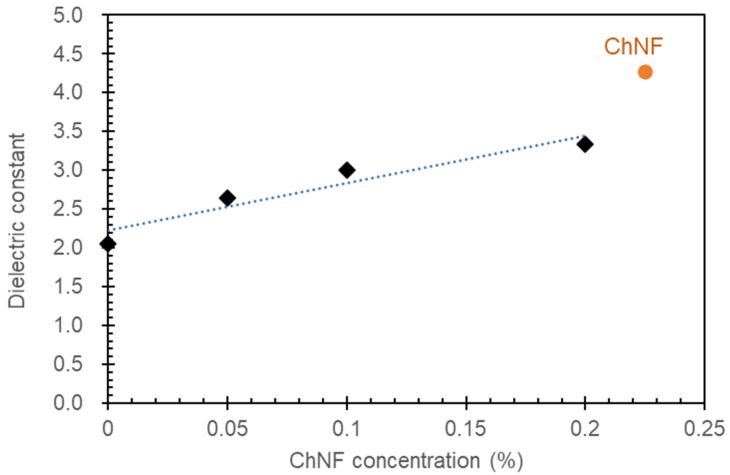
Dielectric constant of the composites as shown by the black diamond marks. The dot line is a fitting curve. The orange circle mark is the dielectric constant of ChNF membrane.

Figure 3 illustrates the relationship between the concentration of ChNF in NRL/ChNF composites and the dielectric constant. As the concentration of ChNF increases, the dielectric constant also rises. The dielectric constant of NRL without ChNF added is approximately 2.0. As the ChNF concentration increases to 0.05, 0.1, and 0.2%, the dielectric constant escalates to approximately 2.6, 3.0, and 3.3, respectively. These results indicate that ChNF significantly influences the dielectric properties of NRL, with higher concentrations of ChNF correlating with increased dielectric constants. The enhancement in dielectric constant can be attributed to the polarizability and microcapacitor effect of ChNF. NRL is non-polar, whereas ChNF is polar due to the presence of hydroxyl and amino groups, which can align in an electric field, contributing to the overall polarization of the composites and, consequently, an increase in the dielectric constant. Additionally, ChNFs within the composite can act as microcapacitors. With a higher content of ChNF, the NRL composites contain more of these microcapacitors, which can store electric charge more effectively, further contributing to an increased dielectric constant. Similar phenomena regarding the tailoring of the dielectric properties of NRL have been observed with other nanofillers, such as zinc oxide, titanium dioxide, nanoclays, carbon nanotubes, graphene, carbon black, and millable polyurethane elastomer [28,29,30,31,32,33].

**Figure 4:**
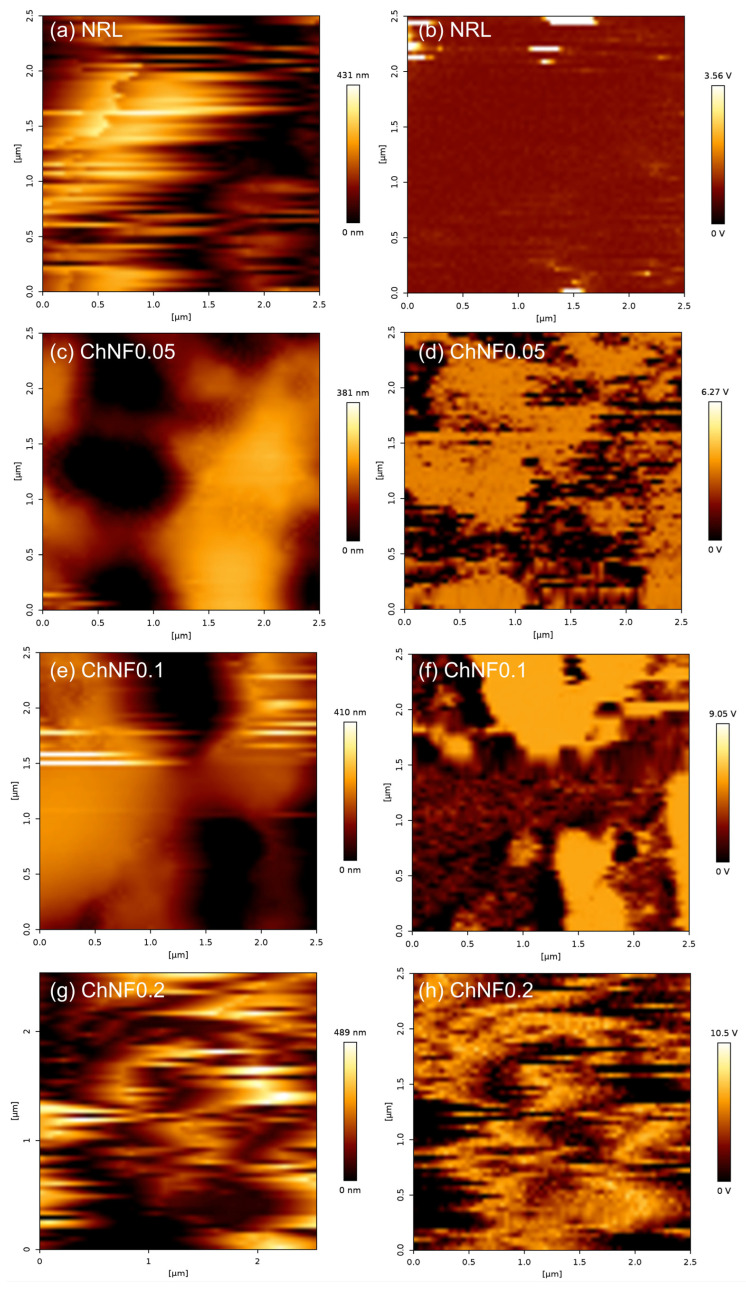
AFM data of the NRL and NRL/ChNF composites. (**a**,**c**,**e**,**g**) Topographaphy. (**b**,**d**,**f**,**h**) Surface potential difference.

**Figure 5:**
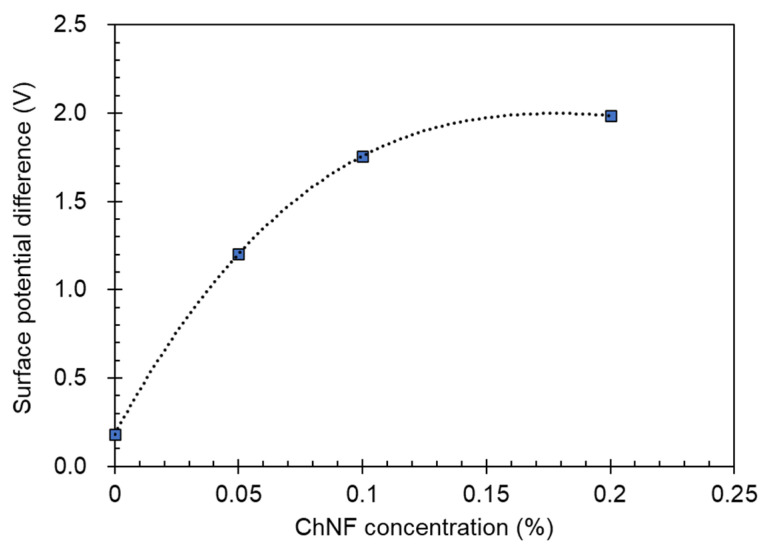
Relationship between surface potential difference and ChNF concentration as shown by the blue square marks. The dot line is a fitting curve.

The significance of surface potential in influencing the electron gain or loss capacity of materials, and consequently the output performance of TENGs, is well established [34]. To assess this, KPFM was employed to measure the surface potential of both pure NRL and NRL/ChNF composites. Utilizing the KPFM mode, the spatial distribution of SPD was mapped, as depicted in Figure 4, which also includes topographical data. Notably, the NRL/ChNF composites exhibited higher surface potential compared to pure NRL. A higher ChNF concentration results in a higher SPD, as shown in Figure 5. This enhancement is attributed to the improved electron-donating capability, resulting from the exposure of amine groups through partial deacetylation [35]. Specifically, the SPD of NRL increased from 0.182 V to 1.987 V in the ChNF0.2 composite. The reinforcing effect of ChNFs on the mechanical properties of NRL/ChNF composites was also evaluated. Remarkably, the ChNFs enhanced the mechanical strength of the NRL; the Young’s modulus value increased from 445 MPa for NRL to 3.4 GPa for ChNF0.2, as revealed in Figure S2. The excellent mechanical performance of the NRL/ChNF composites can be ascribed to the high aspect ratio of ChNFs and their nanoscale dispersion in NRL [36].

**Figure 6:**
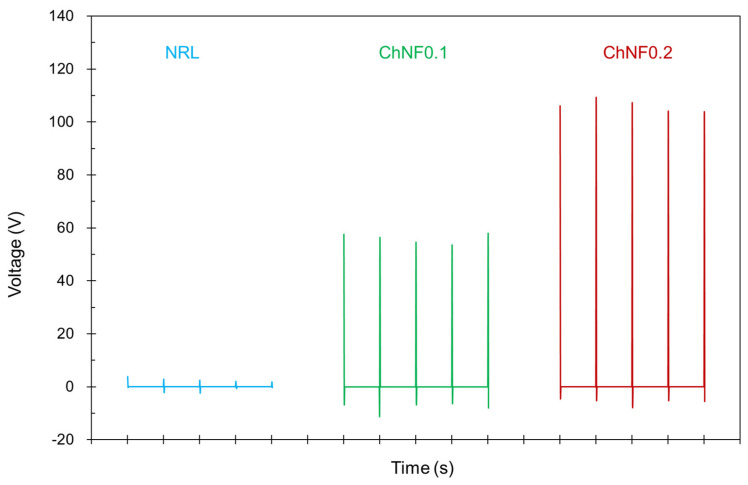
TENG performance of each sample.

Figure 6 presents the output voltage measurements of TENG devices fabricated using different NRL/ChNF composites. The voltage peaks correspond to periodic mechanical actions applied to the TENG devices, which generate the electrical output. A TENG with a triboelectric layer solely comprising NRL exhibits very low voltage outputs, approximately 2.56 ± 0.8 V, indicating that NRL alone may have a relatively low triboelectric effect. However, when ChNF0.1 was used as the triboelectric layer, there was a significant increase in voltage output to approximately 56.04 ± 1.9 V, suggesting that the incorporation of ChNF enhances the triboelectric effect. Furthermore, a triboelectric layer composed of ChNF0.2 yields even higher voltage outputs than ChNF10 to 106.04 ± 2.3 V, implying that the addition of a greater concentration of ChNF continues to improve the triboelectric properties of the composite material. ChNF0.2 shows the maximum output, enabling an approximately 40-fold improvement in output voltage compared to NRL.

The significant enhancement in output voltage observed with NRL/ChNF composites can be attributed to the role of ChNF, which notably contributes to the performance improvement of TENGs through electrification and electrostatic effects. Concerning electrification, the triboelectric output is empirically linked to the relative positions of the materials on the triboelectric series [1,37]. The position of a material on this series correlates with its surface potential. Materials with a high surface potential tend to donate charge (tribo-positive), while those with a low surface potential are more likely to accept charge (tribo-negative). Practically, the greater the disparity in surface potential between two materials, the more enhanced the triboelectric performance of the resultant TENGs. NRL is slightly more positive on the triboelectric series compared to PET [1,37]. NRL/ChNF composites, exhibiting increased surface potential, alter their position on the series. Consequently, TENGs incorporating NRL/ChNF composites and PET demonstrate an amplified electrification effect, leading to improved TENG output. Additionally, in TENG devices employing NRL/ChNF composites as the triboelectric layer, the electrostatic effect is significantly augmented. The integration of ChNF into NRL raises the dielectric constant of the composites, thereby increasing the charge capacitance at the surfaces of the triboelectric materials. This enhancement in charge capacitance results in amplified triboelectric charges, which are instrumental in boosting the electrical output of the TENG.

We have successfully demonstrated the synthesis of NRL/ChNF composites and their application in natural material-based TENGs. However, future research in this domain should concentrate on several critical aspects to further augment the performance and practicality of TENGs employing NRL/ChNF composites. These aspects include enhancing energy-harvesting efficiency, conducting extensive durability studies, and developing specific applications where the distinctive properties of NRL/ChNF composite-based TENGs are optimally utilized. Potential applications encompass wearable electronics, self-powered sensors, and the extraction of energy from ambient mechanical vibrations across various industrial sectors. By focusing on these areas, future research endeavors have the potential to significantly propel the advancement of TENG technology, leading to solutions that are not only more efficient and robust but also environmentally sustainable.

## 4. Conclusions

In this research, we have successfully demonstrated the potential of ChNFs derived from shrimp shell bio-waste as an environmentally sustainable candidate for TENG applications. The HPM process demonstrates efficacy in dispersing and differentiating ChNFs for achieving well-distributed ChNFs with a consistent diameter. By incorporating ChNF into NRL to create NRL/ChNF composite materials with ChNF concentrations of 0.1 and 0.2 wt%, the mechanical properties of NRL were enhanced, as evidenced by the increased Young’s modulus of NRL/ChNF (ChNF0.2) to 3.22 GPa, surpassing that of pure NRL. Furthermore, these composites demonstrated a higher dielectric constant of 3.3 at 0.1 MHz, indicative of their superior charge storage capacity. Moreover, the surface potential difference of NRL increased from 0.182 V to 1.987 V in the NRL/ChNF composite. When applying the NRL/ChNF composites as the triboelectric layer within TENGs, the NRL/ChNF composites yielded a remarkable elevation in electrical output, with a recorded voltage surge up to 106.04 ± 2.3 V. This significant increase in performance is ascribed to the improved dielectric properties of the NRL/ChNF composites. Our findings offer a practical, green synthesis route for advanced natural materials that are well-suited for next-generation energy-harvesting devices, paving the way for broader applications of sustainable energy technologies.

## Data Availability

Data are contained within the article and supplementary materials.

## Supplementary Materials

The following supporting information can be downloaded at: https://www.mdpi.com/article/10.3390/ma17030738/s1. Figure S1. Schematic views of (a) the NRL/ChNF triboelectric layer preparation process (b) TENG device. Figure S2. AFM images of Young’s modulus mapping of (a) NRL and (b) ChNF0.2.
