# Supplementary material for: Preparation of Chitin Nanofibers and Natural Rubber Composites and Their Triboelectric Nanogenerator Applications
Source: Materials (Basel). 2024 Feb 3;17(3):738. doi: 10.3390/ma17030738 (PMC10856660; doi:10.3390/ma17030738)
Supplement: Supplementary file 1 [file materials-17-00738-s001.zip › materials-2733072-supplementary.pdf]

Electronic supplementary information

Preparation of chitin nanofibers and natural rubber composites and their triboelectric nanogenerator applications

Kattaliya Petchnui [1], Teerayut Uwanno [1], Mayuree Phonyiem Reilly [1], Chinathun Pinming [1], Alongkot Treetong [2], Visittapong Yordsri [3], Nutthanun Moolsradoo [4], Annop Klamcheun [2] and Winadda Wongwiriyapan [1,*]

[1] College of Materials Innovation and Technology, King Mongkut's Institute of Technology Ladkrabang, Chalongkrung Rd., Ladkrabang, Bangkok 10520, Thailand; kattaliya.pang@gmail.com (K.P.); teerayut.uw@kmitl.ac.th (T.U.); mayuree.ph@kmitl.ac.th (M.P.R.); chinathun.aron@gmail.com (C.P.)

[2] National Nanotechnology Center (NANOTEC), National Science and Technology Development Agency (NSTDA), 111 Thailand Science Park, Paholyothin Rd., Klong Nueng, Klong Luang, Pathum Thani 12120, Thailand; alongkot@nanotec.or.th (A.T.); annop@nanotec.or.th (A.K.)

[3] National Matal and Materials Technology Center (MTEC), National Science and Technology Development Agency (NSTDA), 111 Thailand Science Park, Paholyothin Rd., Klong Nueng, Klong Luang, Pathum Thani 12120, Thailand; visittay@mtec.or.th

[4] Department of Production Technology Education, Faculty of Industrial Education and Technology, King Mongkut's University of Technology Thonburi, 126 Pracha Uthit Rd., Thung Khru, Bangkok 10140, Thailand; nutthanun.moo@kmutt.ac.th

* Correspondence: winadda.wo@kmitl.ac.th

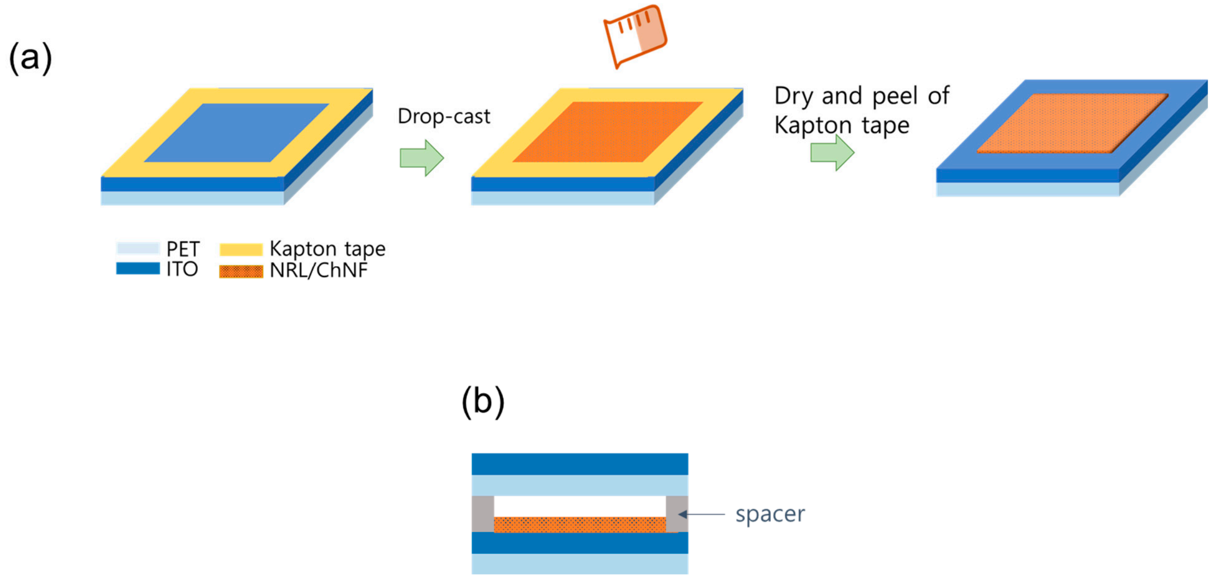

Figure S1. Schematic views of (a) the NRL/ChNF triboelectric layer preparation process (b) TENG device.

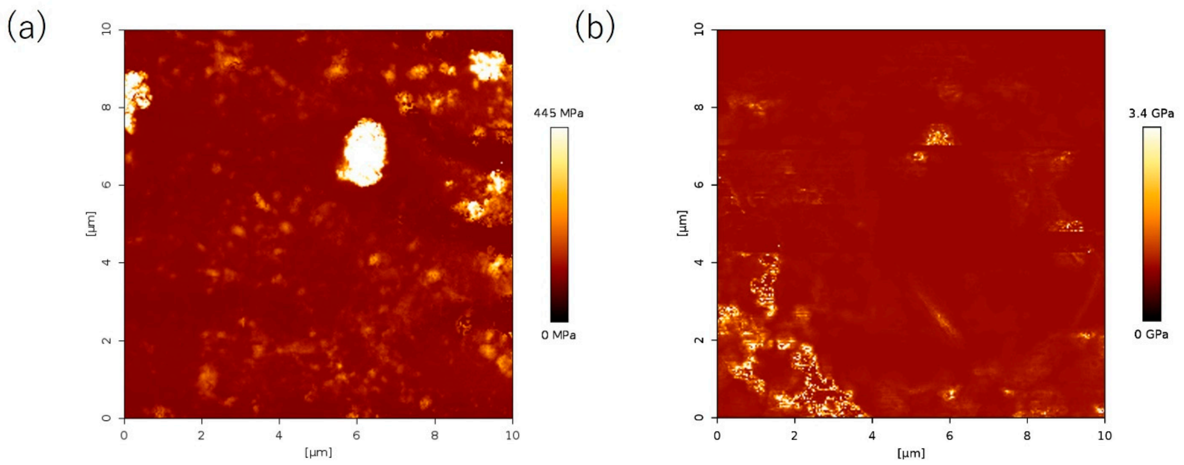

Figure S2. AFM images of Young's modulus mapping of (a) NRL and (b) ChNF0.2
